# The case for citizen science in public health policy and practice: a mixed methods study of policymaker and practitioner perspectives and experiences

**DOI:** 10.1186/s12961-023-00978-8

**Published:** 2023-05-01

**Authors:** Leah Marks, Ben J. Smith, Jo Mitchell, Yvonne Laird, Samantha Rowbotham

**Affiliations:** 1 Menzies Centre for Health Policy and Economics, School of Public Health, Faculty of Medicine and Health, The University of Sydney, Sydney, NSW Australia; 2 The Australian Prevention Partnership Centre, The Sax Institute, Sydney, NSW Australia; 3 Charles Perkins Centre, The University of Sydney, Sydney, NSW Australia; 4 Prevention Research Collaboration, School of Public Health, Faculty of Medicine and Health, The University of Sydney, Sydney, NSW Australia

**Keywords:** Citizen science, Public health, Health policy, Community engagement, Chronic disease prevention

## Abstract

**Background:**

Citizen science (CS) is increasingly being utilised to involve the public in public health research, but little is known about whether and how CS can address the needs of policy and practice stakeholders in health promotion and chronic disease prevention.

**Methods:**

Using a mixed methods approach we conducted an online survey (*n* = 83) and semi-structured interviews (*n* = 21) with policy and practice stakeholders across Australia to explore how CS approaches are perceived and applied in chronic disease prevention, how CS aligns with existing approaches to community engagement, and how the uptake of CS can be supported within policy and practice settings.

**Results:**

Most participants had heard of CS, and while few had experience of using CS, there was widespread support for this approach, with many seeing it as complementary to other community engagement approaches. CS was seen as providing: (a) a robust framework for engagement; (b) access to rich data; (c) opportunities for more meaningful engagement; and (d) a mutually beneficial approach for stakeholders and community members. However, stakeholders identified a need to weigh benefits against potential risks and challenges including competing organisational priorities, resourcing and expertise, data quality and rigour, governance, and engagement.

**Conclusions:**

To expand the use of CS, stakeholders identified the need for increased awareness, acceptance, and capacity for CS within public health organisations, greater access to supporting tools and technology, and evidence on processes, feasibility and impacts to enhance the visibility and legitimacy of CS approaches.

**Supplementary Information:**

The online version contains supplementary material available at 10.1186/s12961-023-00978-8.

## Background

Addressing complex problems in chronic disease prevention hinges on purposeful engagement with all actors that can effect change, including the public. Recent decades have seen a shift in focus away from a deficit model towards harnessing community assets; working ‘with’ rather than ‘for’ communities to promote health and reduce inequities [1, 2]. Public health organisations in Australia and internationally recognise the need for meaningful involvement of citizens, communities and constituencies as key agents in decision making around health and wellbeing [3–5]. For example, pursuit toward ‘collaboration’, ‘co-design’ and ‘co-production’ between government organisations, non-government organisations, communities and individuals have emerged as key pillars of local, state, and national strategic plans [6–8]. However, in practice, organisations have varying capabilities to operationalise this type of engagement [9, 10], and many are looking to innovative and engaging ways of involving the public in addressing complex issues, including citizen science [11–17].

Citizen science (CS) approaches, which seek to actively involve members of the public (known as *“citizen scientists*”)^1^ in scientific research are increasingly being used in public health and chronic disease prevention [19]. Involvement in collecting and analysing data is often a defining characteristic of CS [20–23], but CS can encompass a broad range of activities including involving citizen scientists in shaping research questions through to interpreting findings and prioritising and advocating for change. CS is attracting the interest of policy and practice organisations across a wide range of sectors (e.g. environment, conservation, education, urban planning) due to its potential to generate novel data in a cost-effective way, to engage and empower communities [24–26] and to improve the coherence of policies and programs with community needs [27–29]. A recent scoping review [19] found CS approaches have been used in chronic disease prevention to address a range of issues, including physical activity and green space [30, 31], nutrition and food environments [32–34], mental health [35–37], tobacco and alcohol control [38, 39], work environments [40] and environmental sustainability [41–43].

While still a relatively new area, research has demonstrated the feasibility and utility of CS and its potential to bring about impacts for participants (e.g. improved health and scientific literacy, social connectedness, empowerment) and for policy and practice (e.g. informing priority setting and supporting the design and implementation of actions to address community-identified needs) [34, 35, 38, 44–48]. Despite considerable potential for CS approaches to contribute to addressing the strategic objectives of policy and practice organisations, there has been little documented uptake of these approaches by organisations in public health. For example, a scoping review of CS approaches in chronic disease prevention found most projects to date have been researcher-led with only limited involvement of policy and practice stakeholders [19], while a content analysis of how the term “citizen science” is used in international policy documents identified only one document related to public health [28]. As such, there is little understanding about whether and how CS may be used to address the needs of public health organisations or what these organisations need in order to harness these approaches within their work.

This study aimed to (1) explore policy and practice stakeholders’ perceptions of and experiences with CS approaches in public health, particularly in relation to how stakeholders conceptualise CS relative to their existing practice or see this as a viable approach in their work, and (2) better understand how to support uptake of these approaches in policy and practice settings. This research will inform strategies to enhance the utilisation of CS methods in public health policy and practice.

## Methods

### Research design

This research adopted a sequential explanatory mixed-methods approach to data collection [49, 50], and is reported in line with the guidelines for conducting and reporting mixed methods research [51]. An online survey was conducted in the first phase and semi-structured interviews were conducted with a purposive sub-sample of survey participants in the second phase. The study was approved by the University of Sydney Human Research Ethics Committee (REF: 2020/744). All participants were required to provide informed consent prior to participating in the study.

Throughout this paper we broadly refer to community engagement to mean the range of processes that involve community members in decisions that affect them, including the planning, development, delivery and evaluation of policies, programs and services as well as “activities which aim to improve health or reduce health inequalities” ([52], p. 7), often through information sharing and consultation, and occasionally active involvement. While it can be viewed on the spectrum of community engagement, CS is usually defined in terms of the active involvement of members of the public in the research process, particularly in collecting and analysing data, to address real-world problems[23]. Although we note the term “citizen science” and its definition is the subject of scholarly debate[18, 53, 54].

### Participant recruitment

The recruitment strategy focused on reaching a diverse cross-section of policy and practice stakeholders working in the broad public health field, including those in health, social services, transport, sport and recreation, planning and public spaces and environment sectors, which play a key role in protecting and enhancing liveability and wellbeing of/in communities. Although we note there are growing applications of CS and crowdsourcing approaches in biomedical and infectious diseases research [55, 56], our area of interest in this study focused on health promotion and chronic disease prevention (including research, policies and programs to reduce chronic disease and/or associated risk factors). Participants employed in an organisation with a focus on supporting health and wellbeing and/or creating healthy environments, in a role that involves policy or program planning, management or decision making, and who were 18 years of age or older and spoke English were eligible to participate in this study. We adopt the term ‘policy and practice stakeholders’ in this paper to refer to this diverse group of practitioners, public servants and policy or decision makers including those that work in local councils, government departments or ministries, non-government organisations, and charity organisations.

#### Phase 1: survey

The online survey was advertised via direct emails to individuals known to the researchers, relevant mailing lists and online newsletters (e.g. The Australian Prevention Partnership Centre, Australian Health Promotion Association), and social media posts (Twitter and LinkedIn). We also identified relevant individuals and organisations through internet searching and emailed them directly to invite them to participate and/or to request they distribute the email invitation via their staff networks. To optimize the response rate in direct emails, non-responders were sent a maximum of two email reminders [57].

#### Phase 2: interviews

Survey participants were asked to indicate whether they were willing to take part in a follow-up interview. The selection of interview participants was purposive to ensure variation in participants’ roles, the organisations and sectors in which they worked, as well as their reported familiarity and experience with CS approaches. Of the 36 participants indicating an interest in completing the phase 2 interviews, 28 were followed-up and invited to participate. Two additional participants were recommended by interviewees and asked to complete the survey retrospectively in addition to the interviews.

### Data collection

#### Phase 1: survey

The survey was informed by the literature on community engagement in public health research, policy, and practice [58, 59]. This consisted of 41 questions, and asked participants to report on the frequency, nature and objectives of community engagement and CS activities used, familiarity with CS, perceived challenges of CS and ratings of the value of this approach (Additional file 1). The survey also included open-ended questions which asked about participants’ perspectives of the value of community engagement in their work, what they understood “citizen science” to mean and any experiences with CS. Socio-demographic information collected about participants included age, gender, role, sector and organisation. Two senior health policymakers (including one member of the research team [JM]), piloted the survey for face validity, and the survey was modified for clarity and conciseness based on feedback. The survey was hosted on Qualtrics [60] and conducted between January and October 2021.

#### Phase 2: interviews

A semi-structured interview guide was developed to build upon insights gained through the survey, exploring how stakeholders conceptualise CS approaches and its alignment with their current practice around community engagement, their perceptions of the benefits, opportunities and challenges of these approaches as well as any resourcing and capacity needs to support the use of CS in their work (Additional file 2). Stakeholders with experience using CS approaches were also asked to reflect on how these approaches have been applied within their work, and their feasibility and impacts. The interview guide was pre-tested with a senior policy maker on the research team (JM) and refined through team discussions. Interviews were conducted between May and October 2021 via online videoconferencing (*n* = 20) or telephone (*n* = 1) and averaged 45 min in duration (range 29–82 min). Interviews were conducted by the lead author (LM), who did not have an established relationship with interviewees prior to the study. Interviews were audio recorded and transcribed for analysis. Interviewees were given the opportunity to review their transcripts and provide clarifications prior to data analysis. Interviews concluded once all eligible and consenting participants had participated.

### Data analysis and integration

We used a convergent approach to data analysis [49, 50], where survey and interview data were initially analysed separately, and later integrated in order to address the research aims.

#### Quantitative analysis

Quantitative survey data were exported from Qualtrics into Microsoft Excel and IBM SPSS V.26. to calculate descriptive statistics. We examined quantitative data for relationships between familiarity with and perceptions of CS and contextual and demographic characteristics using SPSS. Demographic categories such as sector and organisational level, and 5-point Likert scale responses were collapsed for the purpose of cross-tabulation and reporting by combining “1–2” and “4–5” responses into two categories and keeping “3” as a neutral response.

#### Qualitative analysis

Qualitative survey data and interview transcripts were de-identified and imported into NVivo qualitative data analysis software [61] and analysed using thematic analysis [62]. Coding categories and concept and theme labels were derived inductively from the data. Two members of the research team (LM and SR) independently coded three transcripts and met to discuss preliminary codes, and then the lead author (LM) coded the remaining dataset and discussed any uncertainties with another team member (SR). A framework to cluster and organise concepts into broader themes and subthemes was developed in consultation with the wider research team and revised iteratively. Text responses collected from open-ended survey questions were initially analysed separately.

#### Integration of the data

Comparison of the qualitative and quantitative datasets began after initial analysis of the survey and qualitative data. In comparing the two datasets, we sought to examine whether our qualitative insights could supplement or elaborate upon our quantitative findings by interrogating any inconsistencies or divergences between these datasets.

Qualitative data obtained from the survey were mapped to the coding framework developed for the interview data to be able to examine any similarities, differences or inconsistencies in themes. No new concepts were identified, and responses were coded to existing themes and subthemes. All team members were involved in the final organisation and presentation of themes.

## Results

We begin with an overview of participant characteristics, before presenting findings related to CS approaches under four main themes: *(1) Conceptualisations of CS and alignment with current approaches; (2) Opportunities for CS in public health; (3) Perceived challenges of CS*; and (4) *Supporting CS in policy and practice*.

### Participant characteristics

A total of 83 people took part in the survey, and the majority were female and aged between 30 and 64 years of age. Most survey participants (*n* = 58, 71%) worked in health promotion and chronic disease prevention, followed by health care (*n* = 9, 11%) and urban planning (*n* = 8, 10%). Over three quarters of the sample (*n* = 50, 79%) worked in government organisations (GO), primarily at state or territory level (*n* = 54, 66%). The characteristics of interview participants was similar to survey respondents. See Table 1 for a more detailed breakdown of participant characteristics.

**Table 1:** Characteristics of study participants

Characteristics^1^	Phase 1 Survey (*n* = 83)		Phase 2 Interviews (*n* = 21)	
	*n*	%	*n*	%
Age group (years)				
18–29	7	8.6%	1	4.8%
30–39	21	25.9%	5	23.8%
40–49	19	23.5%	4	19%
50–64	33	40.7%	10	47.6%
65 or older	1	1.2%	1	4.8%
Total	81	100	21	100
Gender				
Female	64	80%	18	85%
Male	13	16.3%	2	10%
Prefer not to say	3	3.8%	1	5%
Total	80	100	21	100
Sector^2^				
Health promotion and chronic disease prevention	58	60.4%	15	70%
Health care	9	9.4%	1	5%
Planning & public spaces	8	8.3%	2	10%
Transport	5	5.2%	1	5%
Sport, recreation & active transport	5	5.2%	2	10%
Human services and social assistance	4	4.2%	0	0%
Environment	3	3.1%	0	0%
Other	4	4.2%	0	0%
Total	96	100	21	100
Professional role/Occupation				
Program manager	21	26.3%	6	28.6%
Senior policy officer	17	21.3%	5	23.8%
Senior manager/Executive	9	11.3%	1	4.8%
Policy/Program director	9	11.3%	3	14.3%
Policy officer/analyst	5	6.3%	1	4.8%
Dietitian or nutritionist	4	5.0%	2	9.5%
Research officer/manager	3	3.8%	0	0%
Health promotion officer	3	3.8%	0	0%
Other	9	11.3%	3	14.3%
Total	80	100	21	100
Organisation type				
Government organisation (GO)	50	79.4%	16	76.2%
Non-government organisation (NGO), and peak bodies	4	6.3%	1	4.8%
Not for profit (NFP) or charity	3	4.8%	3	14.3%
Other	6	9.5%	1	4.8%
Total	63	100	21	100
Level of organisation^2^				
Local or regional	30	30.3%	6	27.3%
State/Territory	54	54.5%	13	59.1%
National	14	14.1%	3	13.6%
Global	1	1%	0	0.0%
Total	99	100	22	100

^1^Note: a sequential sampling approach was used to recruit participants throughout the two phases of this study. Therefore, participants appear in one or both phases of the study

^2^ Participants had the option of selecting more than one sector in which they work, and level of their organisation

### Conceptualisations of CS and alignment with current approaches

Three quarters of survey participants reported being at least “slightly familiar” with the term “citizen science” (*n* = 62, 75%), including 12% reporting being very familiar (*n* = 10). Most interview participants were familiar with the aims and principles of CS, though terminology used to describe CS was often intertwined with other terms, like consumer and community engagement, community consultation, co-design, co-production and partnership.

*Well, I’ve not really heard the word before—citizen science... Certainly not familiar with the terminology but familiar with the concept of research being collated with members of the public. That’s very, very much kind of done, as much as possible within our organisation.* [P. 42, Senior policy officer, GO]

*Maybe we don't use that terminology as such. We probably just call it co-design or participatory research, I think we've called it, but in essence, it's very much the same thing*. [P.49, Nutritionist, GO]

Participants had often encountered CS through environmental projects they had heard about or participated in, though a small proportion had come across CS through health-related projects.

Participants described several features which set CS apart from current community engagement, including the values they ascribed to CS and practical differences in conducting a CS project. A summary of the defining characteristics of CS that were identified is provided in Table 2.

**Table 2:** The defining characteristics of citizen science

Conducted under a research framework	• Community involvement in scientific research • Involvement in data collection
Nature of involvement	• 'Active' involvement • Involvement throughout projects (e.g. through multiple project phases, or from the outset)
Leadership and governance	• More community-led or bottom-up • Loose or flexible approach
Democratisation of knowledge production and solutions generation	• Community seen as a "genuine stakeholder" • A more democratising and empowering approach

Citizen science was understood in various ways, though the involvement of community members in scientific research, particularly involvement in data collection was commonly discussed as a defining feature of CS, consistent with widely accepted definitions of CS. However, operating under a ‘research framework’, within CS was considered pragmatically different to how many described conducting other engagement activities.

*… [CS is] something that’s quite rigorous that has some, sort of, evidence base behind it, in terms of the methodology. Something that’s been tried and tested and obviously peer reviewed in terms of the methodological approach.* [P.31, Senior policy officer, charity organisation]

The degree of involvement of community members in the research process was a distinction that participants frequently drew on in defining CS approaches. While, for some, CS was articulated purely as a data collection exercise, for others data capture was just one component of a sustained engagement across the course of a project. For example, many participants considered involvement of community from the outset and/or across the whole length of projects as key features of a CS approach, including contributing to identifying research priorities or defining research questions, though to designing research methodologies, conducting projects, and disseminating findings.

Another characteristic used to define CS related to its unique connection to those affected by issues and/or their involvement in contributing to potential solutions. Additionally, some participants described CS as a process of bringing people together to achieve greater collective impact.

*Where the broader population is enabled, generally through technology, in participating in the solution of a problem that would be difficult to tackle by one person alone. Different perspectives and collective power bringing solutions.* [P.37, Health promotion officer, GO, survey]

CS was seen by many to align with their philosophy and approach to community engagement. Several participants considered CS as an extension of their existing practice and when considered on a spectrum of public involvement, CS was seen as a more collaborative and democratising approach. Central to this was a common perception that CS represented a shift in power dynamic and a recognition of the public as a valued and “genuine stakeholder” who’s contribution is unique and instrumental to project success. CS was described as more empowering, bottom-up, and “more equal” collaboration between professionals and community members, when contrasted with community consultation. There were some who already engaged with their communities in ways which aligned with their understanding of CS, which encompassed co-designed, grass roots or community-initiated processes, emphasising a focus on two-way exchange and agency.

*health promotion is … working with us, not on us, that sort of philosophy, that’s health promotion 101. So that is obviously a main aim, is making sure that we are working with people, so that’s the whole co-design collaborative approach that we take, the community-lead approach that we take, the capacity building approach we take. So that is one of the main objectives, working with communities.* [P.82, ‘Other’ occupation, GO]

By contrast, those who described their engagement processes as predominantly stakeholder-led (e.g. inviting public submissions), as well as those who didn’t engage under a research framework or described doing less direct community engagement, tended to see the practice of CS as quite new and/or unfamiliar.

### Opportunities for CS in public health

A substantial proportion of survey (*n* = 39, 47%) and interview (*n* = 7, 33%) participants indicated their organisation engages in activities that could be considered as CS and/or they had been involved in a CS project as part of their work (survey *n* = 36, 43%; interview *n* = 6, 29%). Most interview participants with CS experience worked in health promotion and chronic disease prevention and at state/territory level. Participants described CS projects in the areas of physical activity and walkability, urban development, healthy schools, food security in rural and remote communities and healthy lifestyle programs co-designed with vulnerable communities. For example, one participant described local government-led CS projects that aimed to support people to “advocate for increased active living infrastructure within their neighbourhood” (P.82). Another described a state government-led project, which aimed to engage communities in assessing the quality of green spaces in their neighbourhood and increase community demand for improved built environments.

*We were going through planning reforms, so we really saw this was kind of a watershed opportunity…. So we realized that, when we were doing all of those people in the room, there was a big gap by not having citizens and we were using citizen science strategies as a way of trying to fill that gap, and local government loved it.* [P.83, Policy/Program director, research translation organisation]

**Fig. 1:**
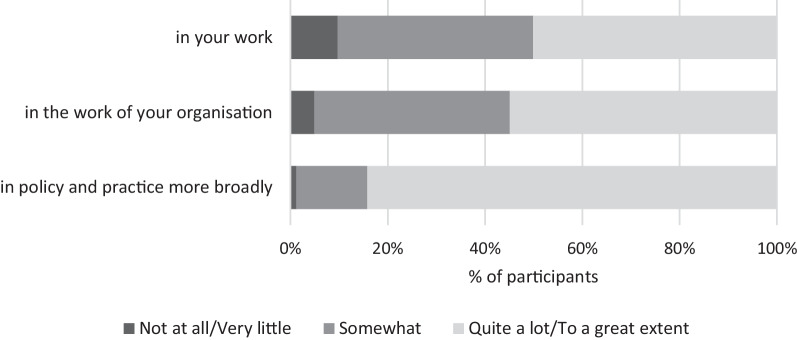
Perceived areas of application for citizen science in the work of participants (*N* = 83)

As shown in Fig. 1, half of survey participants saw a role for CS in their work (*n* = 41, 50%) and the work of their organisation (*n* = 45, 55%), and most saw a role for CS in public health more broadly (*n* = 69, 84%).

Most interview participants saw CS as complementary to their current work and saw CS as a way to address some of the challenges of current approaches to community engagement, which were considered to be overly prescriptive and too disconnected from those affected by the issues they seek to address.

It was against this backdrop that participants perceived the relative advantages of CS approaches in terms of: (a) a more rigorous approach to community engagement, (b) accessing richer data; (c) increasing meaningful engagement; and (d) a mutually beneficial approach.

#### A more rigorous approach to community engagement

Several participants reflected on a history of ad-hoc community engagement and described their past community engagement experiences as ‘box-ticking’ exercises, to fulfill statutory or organisational requirements. By contrast, CS was thought to provide a more robust and systematic research framework around engagement activities, which could lend greater credibility, visibility and rigour to qualitative data like perceptions and experiences that could sometimes be seen as “soft”. Collecting data in a more “evidence-based” way was seen as important to ensure they were receptive to community-identified issues, and to justify decisions that were reached.

*I think [citizen science is] very separate because there’s no, I mean, the way in which we engage is very organic, it’s very – it can be quite ad hoc – it’s not what I call a systematic approach. So obviously that’s very open to biases.* [P.31, Senior policy officer, charity organisation]

#### Accessing richer data

Participants discussed the value of CS in terms of its potential to provide access to new and richer data through capturing more diverse perspectives and lived experiences that cannot be obtained through other means or are considered ‘hard-to-reach’. For example, over two thirds of participants saw value in CS as an approach to improve the relevance of research (*n* = 58, 71%) and evidence-based practice (*n* = 56, 68%) (rated as “*very valuable”* or “*extremely* valuable”), and discussed how CS enables the generation of rich evidence as an important input to decision making.

*I feel that it [CS] just adds value to the work that I do, because I hear perspectives that I'm not going to hear from any other stakeholder around me... And so I feel like when we leave the citizens out or the community out, we very much hear only one part of the story...* [P.49, Nutritionist, GO]

*…it's better data that you collect, because simplistically, you're getting more people to collect it. So you've got more data. You've got a wider variety of perspectives and inputs.* [P.26, Strategic Planner, GO]

For many participants CS was also discussed as a way to “bridge the gap” between population-level data and local insights and lived experiences of issues on the ground. Participants operating at local or regional levels often discussed the challenge of relying on national and state-wide data to inform their decision making, in that this “one size fits all” approach does not account for place-based differences in how health issues affect communities differently.

*I think Citizen science gives us a chance to have more local data. We’re often trying to translate data from [city] at worst, or just from statewide data at best, and it doesn’t always translate to our community in our context... So it gives us an opportunity to find out more about our community.* [P.5, Program manager, GO]

#### Increasing meaningful engagement

The third subtheme spoke to the opportunities CS offered for more meaningful or “less tokenistic” engagement of the public in research and decision-making processes, which was considered a foundation for realising other wide-reaching benefits.

*…often policy makers and prevention people engage with citizens in a really tokenistic manner, but if you're giving them the tools to be able to collect the data, and they've got the decision-making on what bits of data they collect, and where they collect it, then I think that's giving them a bit of power.* [P.83, Policy/Program director, research translation organisation]

Participants often saw CS as a vehicle to raise community awareness about issues (n = 59, 73%), and to build community support or capacity for action (n = 64, 79%). For example, in interviews, participants spoke of CS as a process which not only has the potential to improve health, scientific and policy literacy, but also to foster community ownership over project findings and increase public interest in and advocacy for public health issues.

*So we were seeing the citizen science not so much as a strategy to gather data, although we did want that, it was more as a vehicle to start to create a community movement where the community could actually start to …first of all, be aware of the issue, have a say in what they thought was really important… and then start to demand from the building sector, the government, local government, different outcomes.* [P.83, Policy/Program director, research translation organisation]

Participants also felt that CS offered untapped potential to harness community voice for policy advocacy and emphasised the power of hearing stories direct from those most affected to bolster their advocacy efforts and increase pressure on governments to act. A few participants also spoke about opportunities afforded by CS to contribute to more meaningful and sustainable policy and practice outcomes, through building trust, transparency and long-term relationships between communities, practitioners and policymakers.

*Politically, I think citizen science approaches have real value and real untapped potential in terms of making a real difference in enabling people and information to be heard and received politically that may not usually be harnessed in that fashion.* [P.82, ‘Other’ occupation, GO]

*I actually think, in terms of sustainability and to get real change, it’s [citizen science] incredibly valuable.* [P.42, Senior policy officer, GO]

#### A mutually beneficial approach

Lastly, CS was considered advantageous because it was seen to offer mutual benefit for both organisations and community members, something participants felt existing engagement approaches often lacked.

*I think sometimes members of the community are looking for something to participate, to give to as well, and obviously to help organisations such as ourselves... So I think that’s something that benefits both sides as well… So all these things that can be done better, because there are just more people doing it and participating than we could possibly do by ourselves.* [P. 11, Senior policy officer, charity organisation]

Some participants felt, if practiced well, CS offered a mechanism for more open, two-way and community-led exchange as a contrast to pre-defined stakeholder-led processes, for example to support communities to raise issues of importance to them and to gather and use their own data to shape policy dialogue and advocate for changes that they want to see.

### Perceived challenges of CS

**Fig. 2:**
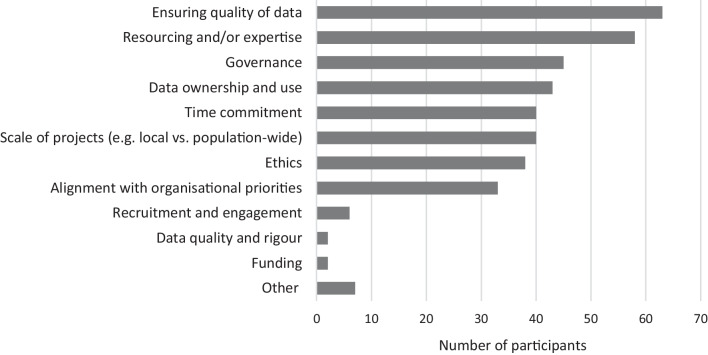
Survey participants’ perceptions of challenges of citizen science approaches (*N* = 82). *Other includes accessibility and applicability of findings; measuring impacts, research design and management, “Sovereignty; fidelity”; and “Over complication of simple community feedback”)

Across survey and interview participants, the main challenges of CS approaches were perceived to be ensuring data quality (*n* = 63, 77%), resourcing and/or expertise (*n* = 58, 71%), project governance (*n* = 45, 55%) and recruitment and engagement of citizen scientists (*n* = 12, 57%) (see Fig. 2 for survey responses).

In terms of resourcing, CS was seen as being potentially more time consuming than other approaches, and the funding and time required to plan for, manage and deliver CS projects, as well as developing the expertise to facilitate this process were key challenges.

*It's time consuming, though I don't see that as a deficit, but organisationally, time does cost money. So it's just a general understanding that it will cost a bit of time and money to invest in this approach. Obviously, I think that is worth it for the end results, but it is a constraint organisationally.* [P.49, Nutritionist, GO]

Some participants raised concerns about the quality of CS findings due to biased or non-representative samples (e.g. due to possibility of CS appealing more to health-conscious individuals), and a lack of methodological rigour, and expressed doubts about how receptive some government organisations may be to trusting the credibility of CS findings. It was acknowledged, however, that in some instances obtaining the views of unreached and diverse participants may be more valuable than representativeness and reliability. Some participants acknowledged that for them, CS can offer one piece of the puzzle to complement other data collection activities.

*I think the biggest challenge that you would face would be the reliability of data and being able to defend that… if you could be concerned that it could be skewed based on a handful of people who might have an alternative motive or who might not understand the concept properly…* [P.74, Senior policy officer, NGO alliance]

Governance, including management of citizen scientists and finding the right balance of control over the research process was another common challenge raised. For example, several considered CS as politically riskier than other engagement approaches, due to a greater investment of resources, less control over who participates and their responsibilities, and the potential to hear unexpected or unwanted findings. As such, some emphasised the importance of safeguards such as setting and managing expectations of participants.

Some participants had concerns about the recruitment and sustained engagement of citizen scientists, particularly those from diverse or vulnerable population groups. For example, two participants relayed difficulties recruiting and retaining citizen scientists to take part in CS projects they have conducted. One participant also felt a reliance on volunteers or community champions was riskier than paid employees and had potential to overburden citizen scientists.

*Those projects are vulnerable to falling over because you’re often relying on volunteers or community champions who move on. There’s always the risk of them falling over or not being successful or not being sustainable. But those that do kind of have the right ingredients and go, they’re gold.* [P.18, Policy/Program director, GO]

### Supporting CS in policy and practice

**Fig. 3:**
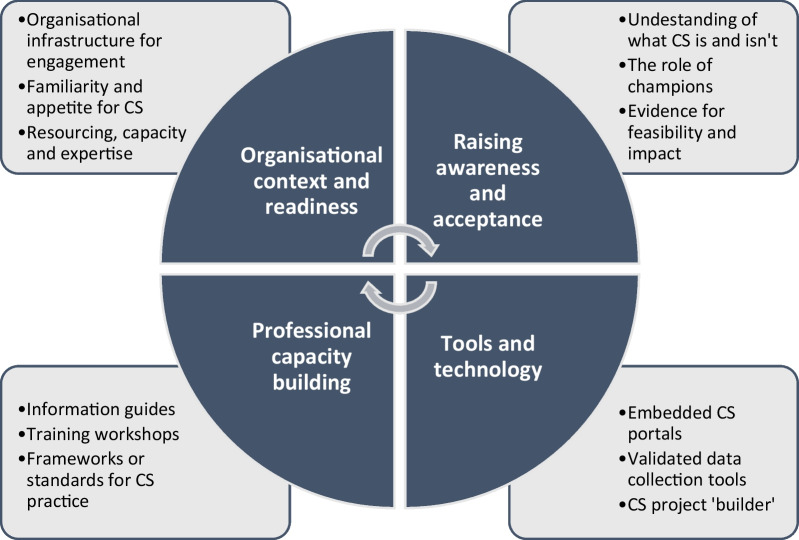
Policy and practice stakeholder requirements to support their use of citizen science approaches

Participants identified various structures and processes to support wider uptake of CS in policy and practice, including the organisational context and readiness, awareness and acceptance, professional capacity building and tools and technology (Fig. 3).

#### Organisational context and readiness

The organisational context, including organisational priorities around community engagement, research agenda and infrastructure, and appetite for CS were considered key factors in influencing the extent to which participants felt their organisations were ready to utilise these approaches. Acknowledging the significant time and costs these approaches can take, many participants highlighted the need for dedicated funding and personnel to support CS activities. Others spoke of existing infrastructure, capacity and expertise to support community engagement activities, including established research and partnership agreements as necessary ingredients.

*Well, I think it needs to be somebody's job. You know, like if we truly want, and I think citizen science is a tool, I think we need a broader kind of policy framing...* [P.83, Policy/Program director, research translation organisation]

Several participants considered CS to be an opportunity to truly realise organisational priorities and/or legislative requirements around increasing consumer involvement in decision making and advocacy. Some discussed CS approaches as almost inevitable or “part and parcel” of how organisations operate in future, as organisations become increasingly consumer-centric.

*I think increasingly as we move forward, consumer engagement and citizen involvement is going to become, a given. It’s going to be part and parcel of how government does business, and it’s already like that. But they’re going to expect, I think a higher level of, in terms of the quality of what’s being offered to them, they’re going to expect a lot more. And I think we, as organisations, have to be attuned to that and have skills to actually accomplish that.* [P.31, Senior policy officer, charity organisation]

However, several participants also expressed scepticism about how CS would ‘fit’ in their organisation’s workplan, which some attributed to a lack of experience in using CS. Others emphasised to achieve many of the benefits discussed, being open, flexible and sharing power with communities was fundamental, but discussed organisational constraints and risks associated with this, including insufficient infrastructure to support meaningful community involvement or a lack of will for institutional change.

#### Raising awareness and acceptance of CS

Almost all interviewees discussed a lack of awareness and understanding about CS within their organisation and considered awareness raising as the critical first step in generating wider acceptance and interest. Gaining clarity about what CS is, how it is distinct from current engagement approaches and what it offers over and above other approaches was considered important in demonstrating the business case for CS.

Participants emphasised the importance of evidence and examples that demonstrate the process, feasibility and outcomes of CS projects, and champions within organisations who could advocate for the use of these approaches.

*Case studies like that done in other governments would be so valuable. I think a lot of it is just trying to work out where the benefits are and if you see similar implementation elsewhere that’s been successful, that’s half your work for you, in terms of trying to get change and trying to start something new.* [P.36, Senior policy officer, GO]

#### Professional capacity building

Participants described a need for information, guidelines and training workshops focussed on the process of designing, implementing, and evaluating CS projects to enable their use in policy and practice settings. Alongside capacity building activities, two participants felt frameworks or standards for the practice of CS could be established and embedded within reporting structures for organisations with mandatory engagement requirements.

*I think one thing that really helped initially for me when I was trying to advocate for this type of approach in our own research was having guidelines…* [P.49, Nutritionist, GO]

#### Technology

Finally, for CS to be more widely adopted, several people spoke of the need for greater access to supporting technology and tools, including affordable and validated data collection tools and an online portal to provide access to current projects and resources to support stakeholders to effectively design a project to address their needs.

*The tools that are available will need to be varied depending on the need they need to serve of course. But the tools out there vary in terms of… their availability and in terms of their affordability and that is definitely something that needs addressing...* [P.82, 'Other’ occupation, GO]

## Discussion

There is considerable potential for CS approaches to support policy and practice goals in public health and chronic disease prevention. While researchers have begun to investigate policy makers’ perspectives of CS[63], to our knowledge this is the first study to explore how CS is perceived and applied in public health policy and practice, with a particular focus on examining how CS was seen to align with existing approaches to community engagement. There was a plurality of understandings about CS that were expressed, and individual’s orientation and approach to community engagement often influenced how they perceived the relative advantages and uses of CS. Given the natural alignment between CS and approaches such as community-based participatory research and participatory-action research[38, 64–66], as well as the numerous definitions and typologies of CS[23, 54, 67, 68], we were unsurprised to find that CS was understood in a wide variety of ways and occasionally overlapped with terminology for other engagement approaches. This mirrors the ‘plurality’ described by Haklay and colleagues[69] and other CS scholars[53, 70–72] in characterising CS as an adaptable approach, that is defined in relation to the context in which it is applied and the functions it is intended to serve. There was a sense among our participants that CS had familiar roots and aligned in principle with many values and aims of their community engagement activities, but would differ in practice from how they typically involve communities in their work. Our findings indicate that in a public health context, greater awareness and adoption of CS may be facilitated by clarification of the conceptual and practical boundaries between this approach and the various methods of community engagement that are used.

We found there was substantial appetite for CS among policy and practice stakeholders working in a variety of government, non-government and not for profit organisations, and those operating at local, regional, state and national levels. CS was seen to offer a range of advantages, with potential to address multiple strategic objectives of organisations. Perceived benefits included providing a framework for more rigorous engagement, contribution of valuable data, meaningful engagement and community mobilisation to achieve sustainable public health outcomes, and offering a mutual “win–win” for citizen scientists and supporting organisations. Participants felt CS may be particularly useful for generating new data in areas which were under-studied and under-resourced, including the evaluation of interventions that have diverse grass roots impacts (e.g. physical activity infrastructure), and investigation of commercial determinants of health (e.g. youth access to vaping and e-cigarettes). This suggested the drivers for using CS in public health were consistent with other policy areas (e.g. environmental, science and planning policy) related to the generation of evidence, provision of resources and civic empowerment[24, 26, 29] albeit with additional goals related to harnessing community voice for policy advocacy. However, further work is needed to examine the motivations and objectives of public health organisations which have used these approaches and how they have gone about translating CS principles into practice in ways which attend to the needs and priorities of community, academic and policy stakeholders.

Ultimately the appraisal of CS by participants in this study was a trade-off between multiple benefits (or “multiple agendas”) and a number of perceived risks. While many participants spoke passionately about what CS could bring to their work and/or that of their organisations, several were sceptical of what CS could offer beyond current approaches or if certain challenges weren’t overcome. Common concerns regarding the resource-intensive nature of CS along with challenges in demonstrating impacts of CS activities, presented barriers to the use of these approaches. Interestingly, CS was considered to offer a more robust method of community engagement, but is also vulnerable to being mistrusted due to issues like validity and generalisability of data. This highlights the need to develop procedures and resources to improve the scientific strength of CS in line with similar approaches[73]. For example, as Chapman and colleagues[27] discuss, for CS to be considered a legitimate tool for supporting decision making and policy development, the evidence produced would need to confer confidence that it was generated by rigorous and appropriate methods. Further leveraging CS at the organisational level necessitates professional development in the design of projects which ‘strategically considers’ and complements the organisation’s needs and existing efforts to maximise the impact of these approaches [74](p.10). Lessons may be learnt from the broader participatory research (PR) and community engagement literature regarding supporting and evaluating engagement activities of this nature in policy settings[9, 75]. For example, in their critical review of PR in public health, Cargo and Mercer[76] argues the advancement of PR science and practice requires strategic investment in four areas: future research which ‘establishes the effectiveness of PR in achieving health outcomes’; funding for infrastructure to support new and ongoing PR projects and to integrate PR into organisations’ operating procedures; education and training in these approaches, and; increased institutional support and policies that recognise the unique nature and benefits of PR as well as their ethical review requirements (p.342). In Australia, research funding programs, including by the National Health and Medical Research Council (NHMRC) are increasing their focus on CS[77]. Greater efforts are also being made to build capacity in CS generally[78], as well as in public health policy and practice[79]. We would therefore expect to see ongoing development of CS methods over the next decade and increasing interest in understanding the contexts in which CS approaches may be applied as they become increasingly salient in public health and politically viable for the policymakers and practitioners who champion them.

### Strengths and limitations

A major strength of this study was the methodological triangulation provided through mixing of survey and interview methods[80], which enabled us to generate two complementary datasets that afforded both diversity in types of participants reached and richness and meaningfulness in results generated. The diversity of our sample who worked in a range policy and practice settings across the broad public health field also bolstered the potential representativeness of findings.

However, the small sample of 83 survey participants was a limitation in this study as it meant we needed to rely on descriptive data rather than using statistical tests to examine relationships between participant characteristics (e.g. sector, organisation type, organisation level) and their familiarity with and perceptions of CS. While we note our interview sample was less diverse than our survey sample, our qualitative analysis of interview data did not surface any clear patterns between participant characteristics and their conceptualisations and perspectives of CS.

## Conclusion

This study demonstrated widespread interest and appetite for the use of CS approaches as an input to inform and shape policy and practice in public health. While the terminology of CS may be relatively unfamiliar, we found the principles and to some extent the practice of CS are known to policy and practice stakeholders and may complement how they engage with the public in their work. To expand the use of CS approaches and achieve their full potential in promoting meaningful community engagement in public health, strategic investment is needed to generate awareness and acceptance of CS approaches and to build capacity, infrastructure, and practical tools to support CS within public health organisations.

## Supplementary Information

**Additional file 1.** Survey tool.

**Additional file 2.** Interview topic guide.

## Data Availability

The datasets used and/or analysed during the current study are available from the corresponding author on reasonable request.

## Abbreviation

CS: Citizen science
PR: Participatory research
GO: Government organisation
NGO: Non-government organisation
NFP: Not for profit organisation

## Footnotes

1 In this paper, we use the term “citizen” to distinguish lay members of the public from professional researchers and do not intend to reflect the citizenship status of the people participating in the research process.(18).
