# Supplementary material for: The case for citizen science in public health policy and practice: a mixed methods study of policymaker and practitioner perspectives and experiences
Source: Health Res Policy Syst. 2023 May 1;21:31. doi: 10.1186/s12961-023-00978-8 (PMC10152701; doi:10.1186/s12961-023-00978-8)
Supplement: Supplementary file 1 — Additional file 1. Survey tool. [file 12961_2023_978_MOESM1_ESM.pdf]

Understanding perceptions of citizen science in policy and practice

Consent to participate

In agreeing to take part in this study I state that:

- I understand the purpose of the study, what I will be asked to do, and any risks/benefits involved.
- I have read the Participant Information Sheet and have been able to discuss my involvement in the study with the researchers if I wished to do so.
- The researchers have answered any questions that I had about the study and I am happy with the answers.
- I understand that being in this study is completely voluntary and I do not have to take part. I understand that I can choose not to answer any questions that I do not wish to answer.
- I understand that I can withdraw from the study at any time, up until the results have been analysed by contacting the researchers.
- I understand that personal information about me that is collected over the course of this project will be stored securely and will only be used for purposes that I have agreed to. I understand that information about me will only be told to others with my permission, except as required by law.
- I understand that the results of this study may be published, and that publications will not contain my name or any identifiable information about me.

○ I agree to take part in this survey (1)

○ I do not agree to take part (2)

THE UNIVERSITY OF SYDNEY

The Australian Prevention Partnership Centre
Systems and solutions for better health

Eligibility criteria

Q1 **Does your organisation have a focus on health and wellbeing, for example, health promotion, healthy environments or preventive health?**

- ☐ Yes (1)
- ☐ No (2)

Q2 **Do you have a role that includes policy or program planning, management or decision making?**

- ☐ Yes (1)
- ☐ No (2)

THE UNIVERSITY OF SYDNEY

The Australian Prevention Partnership Centre
Systems and solutions for better health

Perceptions of public engagement

In this section we ask a few questions about your experiences with public engagement, including how it fits in the context of your work and your organisation's values, priorities and workplan.
Within this survey we use the term "public engagement" to refer to ***deliberate strategies for involving members of the public in research and policy processes***. .

Q4 **To what extent is public engagement considered a priority within your organisation?**

1. Not a priority
2. Low priority
3. Medium priority
4. High priority
5. Essential

Q5 **To what extent does your organisation engage with members of the public in practice?**

1. Not at all
2. Very little
3. Somewhat
4. Quite a lot
5. A Great deal

Q6 **What are the main objectives of public engagement activities in your organisation?**
(*Please select all boxes that apply*)

- ☐ To understand community perspectives and/or needs
- ☐ To help identify priorities and agenda setting
- ☐ To identify solutions
- ☐ To conduct research
- ☐ To raise public awareness or understanding of specific issues
- ☐ To monitor or evaluate policy or programs
- ☐ To contribute to policy or program design
- ☐ To obtain feedback about strategy, policy, programs etc.
- ☐ To increase public support for actions
- ☐ To build public trust, accountability or transparency
- ☐ To pilot resources or communications

- ☐ To promote behaviour change
- ☐ To build community capacity
- ☐ Other (please specify ____________)

Q7 **How frequently does your organisation use the following methods to engage the public?**

	1 - Not at all	*2 - Very little*	*3 - Somewhat*	*4 - Quite a lot*	*5 - A great deal*
Questionnaires					
Focus groups					
Workshops					
Civic dialogues					
Consultations					
Inviting public submissions					
Citizens' assembly or panels					
Citizens' juries					
Community-based participatory research					
Participatory action research					
Citizen science					
Crowdsourcing					
Advisory committees					
Other (please specify ____________)					

Q8 **How frequently does your organisation engage members of the public for the following purposes?**

	1 - Not at all	*2 - Very little*	*3 - Somewhat*	*4 - Quite a lot*	*5 - A great deal*
Identifying or defining issues to be addressed					
Identifying questions that need to be answered through research					
Designing research methods					
Recruiting other participants					
Collecting data					
Analysing data and/or forming conclusions based on the data					
Brainstorming potential solutions					
Disseminating information					

Q9 **How would you rate the value of public engagement for your work?**

- ☐ Not valuable at all (21)
- ☐ Slightly valuable (22)
- ☐ Somewhat valuable (23)
- ☐ Very valuable (24)
- ☐ Extremely valuable (25)

Q10 **What do you see as the main value of public engagement in your work?**

____

<u>Citizen science approaches</u>

Q11 **Are you familiar with the term "citizen science"?**

- ☐ Not familiar at all (30)
- ☐ Slightly familiar (31)
- ☐ Moderately familiar (32)
- ☐ Very familiar (33)
- ☐ Extremely familiar (34)

Q12 **What do you understand "citizen science" to mean?**

____

____

THE UNIVERSITY OF SYDNEY

The Australian Prevention Partnership Centre
Systems and solutions for better health

Perceptions of citizen science

In this section, we will ask about your perceptions of and experiences with citizen science approaches, including whether and how they are used in your work.

Within this survey, we define "**citizen science**" as a broad approach to public engagement that **actively involves** members of the public as collaborators in scientific research (e.g. in collecting and analysing data, identifying research questions, brainstorming solutions and advocating for actions). Members of the public who participate in citizen science are known as citizen scientists.

Q13 **Does your organisation engage in activities consistent with the definition of citizen science above?**

- ☐ Yes (23)
- ☐ No (24)

Q14 **How often does your organisation use these approaches (whether directly, or through third parties)?**

- ☐ Never
- ☐ Rarely
- ☐ Sometimes
- ☐ Often
- ☐ Always

Q15 **Does your organisation use the term "citizen science" to refer to these activities?**

- ☐ Yes (23)
- ☐ No (24)

Q16 **If no, what terms are used to refer to these approaches?**

____

Q17 **What are the main objectives of citizen science activities in your organisation?**
(*Please select all boxes that apply*)

- ☐ To understand community perspectives and/or needs (227)
- ☐ To help identify priorities and agenda setting (228)
- ☐ To identify solutions (229)

- ☐ To conduct research (230)
- ☐ To raise public awareness or understanding of specific issues (231)
- ☐ To monitor or evaluate policy or programs (232)
- ☐ To contribute to policy or program design (233)
- ☐ To obtain feedback about strategy, policy, programs etc. (234)
- ☐ To increase public support for actions (235)
- ☐ To build public trust, accountability or transparency (236)
- ☐ To pilot resources or communications (237)
- ☐ To promote behaviour change (238)
- ☐ To build community capacity (239)
- ☐ Other (please specify) (240) ____

Q18 **Have you been involved in a citizen science project(s) as part of your work?**

- ☐ Yes (23)
- ☐ No (24)

Q19 **Please provide a brief description of the project(s) you have been involved in**

____

____

____

Opportunities for citizen science in your work

In this section we will ask about your perspective of the benefits, opportunities and challenges of citizen science approaches, including whether and how you perceive these approaches as valuable in your work and the work of your organisation (presently and in future).

Q20 **To what extent would citizen science be a valuable approach to public engagement within your organisation?**

- ☐ Not valuable at all (21)
- ☐ Slightly valuable (22)
- ☐ Somewhat valuable (23)
- ☐ Very valuable (24)
- ☐ Extremely valuable (25)

Q21 **To what extent do you think citizen science approaches might be a useful way to...**

	1 - Not at all useful	*2 - Slightly useful*	*3 - Somewhat useful*	*4 - Very useful*	*5 - Extremely useful*
Increase public understanding about issues					
Generate useful data					
Increase public support for policy or programs					
Build public trust, accountability					
Build community capacity for action					
Improve the relevance of research					
Improve policymaking					
Improve evidence-based practice					

Q22 **How would you rate your confidence in the findings of scientific research when....**

	1 - Not at confident	*2 - Slightly confident*	*3 - Somewh at confident*	*4 - Very confident*	*5 - Extremel y confident*
The data have been collected by citizen scientists, but the overall project was led by professional researchers?					
Citizen scientists and professional researchers both contributed to the project design, data collection, and analysis of the data?					
Citizen scientists were completely responsible for the project design, data collection, and analysis of the data?					

Q23 **What do you see as the main challenges of citizen science approaches?**
(*Please select all boxes that apply*)

- ☐ Ensuring quality of data (13)
- ☐ Ethics (14)
- ☐ Resourcing and/or expertise (15)
- ☐ Governance (e.g. lack of control over the research process) (16)
- ☐ Alignment with organisational priorities (17)

- ☐ Time commitment (18)
- ☐ Data ownership and use (19)
- ☐ Scale of projects (e.g. local vs. population-wide scale) (20)
- ☐ Other (please specify) (21) ____

Q24 **To what extent do you think there is a role for citizen science...**

	1 - Not at all	*2 - Very little*	*3 - Somewhat*	*4 - Quite a lot*	*5 - To a great extent*
in your work					
in the work of your organisation					
in preventive health policy and practice more broadly					

THE UNIVERSITY OF SYDNEY

The Australian Prevention Partnership Centre
Systems and solutions for better health

Background information

This section asks a few questions about you and the nature of your work.

Q25 **What is your age group?**

- ☐ 18 - 29 (108)
- ☐ 30 - 39 (109)
- ☐ 40 - 49 (110)
- ☐ 50 - 64 (111)
- ☐ 65 or older (112)

Q26 **How would you describe your gender?**

- ☐ Male (35)
- ☐ Female (36)
- ☐ Other (please specify) (37) ____
- ☐ Prefer not to say (38)

Q27 **What is your workplace postcode?**

____

Q28 **In which sector do you work?**

- ☐ Health promotion and chronic disease prevention (69)
- ☐ Health Care (70)
- ☐ Human services and Social Assistance (71)
- ☐ Transport (72)
- ☐ Environment (73)
- ☐ Planning & Public spaces (74)
- ☐ Other (please specify) (75) ____

Q29 **In which organisation do you work?** *(optional)*

____

Q30 **At what level of policy or practice do you work?**

- ☐ Local or regional (21)
- ☐ State/Territory (22)
- ☐ National (23)
- ☐ Other (please specify) (24) ____

Q31 **What is your occupation?**

- ☐ Policy officer/analyst (66)
- ☐ Senior policy officer (67)
- ☐ Program manager (68)
- ☐ Research officer/manager (69)
- ☐ Policy/Program Director (70)
- ☐ Senior manager/Executive (71)
- ☐ Other (please specify) (72) ____

Q32 **Please briefly describe your role and the focus of your work:**

____

____

____

____

____

THE UNIVERSITY OF SYDNEY

The Australian Prevention Partnership Centre
Systems and solutions for better health

Follow up

Q33 **Would you be willing to be contacted to explore your responses in more depth via a follow-up interview?**

- ☐ Yes (28)
- ☐ No (29)

Q34 **Would you like to receive a summary of the findings from this project via email?**

- ☐ Yes (28)
- ☐ No (29)

Q35 **Would you be interested in finding out more about citizen science approaches?**

- ☐ Yes (28)
- ☐ No (29)

Q36 **What aspects of citizen science would you be interested in learning more about?**
(Please select all boxes that apply)

- ☐ Examples of previous projects (107)
- ☐ Developing citizen science projects (108)
- ☐ Recruitment and engagement with citizen scientists (109)
- ☐ Evaluating citizen science projects (110)
- ☐ Communicating the findings of citizen science projects (111)
- ☐ Citizen science as a method to build community support for preventive health (112)
- ☐ Other (please specify) (113) ____

Q37 **Would you be interested in participating in a community of practice on citizen science approaches in preventive health?**

- ☐ Yes (28)
- ☐ No (29)

Q38 **Please provide us with your name and contact details below:**
Your contact details will be stored separately from your survey responses to ensure your anonymity is maintained.

Name

____

Email address

____

Phone number

____
