# Supplementary material for: The case for citizen science in public health policy and practice: a mixed methods study of policymaker and practitioner perspectives and experiences
Source: Health Res Policy Syst. 2023 May 1;21:31. doi: 10.1186/s12961-023-00978-8 (PMC10152701; doi:10.1186/s12961-023-00978-8)
Supplement: Supplementary file 2 — Additional file 2. Interview topic guide. [file 12961_2023_978_MOESM2_ESM.pdf]

Understanding perceptions of citizen science in policy and practice

Interview topic guide

Topic: Professional role and background	
**Topic**	**Questions**
Position and nature of work<br><br>Public engagement	• I'd like to start by asking you to briefly describe your role and the focus of your work?<br>• To what extent is public engagement a priority within your organisation?<br>a. In what ways does your organisation seek to engage the public?<br>b. For what purposes?
**Topic: Familiarity and experiences with citizen science**	
Familiarity with citizen science<br><br>Understanding of citizen science	• How familiar are you with citizen science approaches?<br>• What do you understand the term 'citizen science' to mean?<br>a. How would you characterise a citizen science approach?<br>b. What are its key features?<br>• In your understanding, how do citizen science approaches differ from other ways of engaging the public?
Alignment	• To what extent do you see citizen science approaches aligning with the work that you do?<br>• Do you see potential for these approaches to be taken up by your organisation?<br>a. What do you think citizen science approaches could contribute to the work of your organisation?<br>• Do you see a role for citizen science within your own work?<br>a. What might that include? E.g. what kinds of projects? For what purposes?<br>b. What could a citizen science approach add?
Experiences with citizen science	<u>If participant answered 'yes' to Q.9 in survey:</u><br>*Organisation level*<br>• You indicated in the survey that your organisation has used citizen science approaches. Can you tell me a bit more about this?<br>a. What types of citizen science projects have been conducted?<br>b. How was this project perceived?<br><br><u>If participant answered 'yes' to Q.10. in survey:</u><br>*Individual level*

	• I would like to hear more about your experiences with citizen science approaches. How have you been involved in citizen science in your work?<br>a. What was the aim of the project(s)?<br>b. What did taking a citizen science approach bring to this project that other approaches may not have?<br>c. Were there any policy or practice impacts as a result of this project? (e.g. impacts on decision making, actions)<br>d. In your experience what factors contribute to successful citizen science project?
**Topic: Perceptions of citizen science**	
Value, benefits and impacts	• What do you see as the main value of citizen science approaches?<br>a. What do you see as the potential benefits for citizen scientists?<br>b. For your organisation?<br>c. For preventive health policy or practice more broadly? (e.g. to understand community perspectives and/or needs, to help identify priorities and agenda setting, to identify solutions, to build community capacity for action, increase public support etc.)
Challenges and limitations	• What do you see as the key limitations of citizen science approaches?<br>a. How about from the perspective of your organisation, are there limitations that your organisation would see as problematic?<br>• What do you see as the main challenges of using citizen science approaches?<br>a. For example, barriers may include data quality, ethics, feasibility issues, expertise, governance, cost of investing in something new etc.<br>b. How do these challenges compare to other public engagement approaches?
Data, resourcing and capacity needs	• What information, resources or support would assist you or your colleagues to use citizen science approaches in your work?
**Topic: Opportunities for citizen science in policy and practice**	
Opportunities for citizen science	• What do you see as being the key opportunities for citizen science in prevention?<br>a. For example, do you think there are opportunities for citizen science as a way to access new data?<br>b. As a way of including different perspectives in decision-making.<br>c. As a way to increase public support?<br>d. As a way to monitor implementation or evaluate policy and programs?

	• In what ways do you think citizen science approaches could be helpful to address current challenges and priorities in prevention or health promotion?<br>a. Can you give any examples?
Citizen science and COVID-19	• Reflecting on the current public health crisis brought on by COVID-19, do you see a role for citizen science to play in assisting public health practice during this time?<br>a. What do you envision this to include?<br>b. Is there a specific gap you think the public may have been able to fill?
**Topic: Closing**	
Closing	• Given that our interest is in strengthening the use of citizen science approaches in policy and practice in prevention, is there anything else you can tell me?<br>• Is there anyone else you think we should talk to about this project? Who?<br>• Do you have any questions for me?
Thank you immensely for your time and your important contribution to this research.	
